# Cross-Regulation between N Metabolism and Nitric Oxide (NO) Signaling during Plant Immunity

**DOI:** 10.3389/fpls.2016.00472

**Published:** 2016-04-08

**Authors:** Elise Thalineau, Hoai-Nam Truong, Antoine Berger, Carine Fournier, Alexandre Boscari, David Wendehenne, Sylvain Jeandroz

**Affiliations:** ^1^Agroécologie, AgroSup Dijon, CNRS, INRA, Université Bourgogne Franche-ComtéDijon, France; ^2^Institut Sophia Agrobiotech, UMR, INRA, Université Nice Sophia Antipolis, CNRSSophia Antipolis, France

**Keywords:** nitrogen metabolism, plant immunity, *Aphanomyces euteiches*, *Medicago truncatula*, nitric oxide homeostasis

## Abstract

Plants are sessile organisms that have evolved a complex immune system which helps them cope with pathogen attacks. However, the capacity of a plant to mobilize different defense responses is strongly affected by its physiological status. Nitrogen (N) is a major nutrient that can play an important role in plant immunity by increasing or decreasing plant resistance to pathogens. Although no general rule can be drawn about the effect of N availability and quality on the fate of plant/pathogen interactions, plants’ capacity to acquire, assimilate, allocate N, and maintain amino acid homeostasis appears to partly mediate the effects of N on plant defense. Nitric oxide (NO), one of the products of N metabolism, plays an important role in plant immunity signaling. NO is generated in part through Nitrate Reductase (NR), a key enzyme involved in nitrate assimilation, and its production depends on levels of nitrate/nitrite, NR substrate/product, as well as on L-arginine and polyamine levels. Cross-regulation between NO signaling and N supply/metabolism has been evidenced. NO production can be affected by N supply, and conversely NO appears to regulate nitrate transport and assimilation. Based on this knowledge, we hypothesized that N availability partly controls plant resistance to pathogens by controlling NO homeostasis. Using the *Medicago truncatula/Aphanomyces euteiches* pathosystem, we showed that NO homeostasis is important for resistance to this oomycete and that N availability impacts NO homeostasis by affecting *S*-nitrosothiol (SNO) levels and *S*-nitrosoglutathione reductase activity in roots. These results could therefore explain the increased resistance we noted in N-deprived as compared to N-replete *M. truncatula* seedlings. They open onto new perspectives for the studies of N/plant defense interactions.

## Nitrogen and the Plant Immune Response

Plants are under the constant threat of pathogen attacks that limit their survival and are major yield-limiting factors. In response to these attacks, plants activate multiple defense reactions both at the site of infection and systemically, which in many cases lead to resistance. These reactions include massive transcriptional reprogramming, cell wall reinforcement, synthesis of antimicrobial metabolites, and production of pathogenesis-related (PR) proteins. These events are mediated by a variety of rapidly mobilized molecules, such as second messengers, e.g., Ca^2+^, protein kinases, reactive oxygen species (ROS), or reactive nitrogen species (RNSs), including nitric oxide (NO). Although these defense responses have been widely studied, it has become increasingly obvious over the past years that a plant’s capacity to mobilize them is greatly affected by its physiological status (Snoeijers et al., 2000) and its development (Develey-Riviere and Galiana, 2007).

Nutrients are important for the growth and development of plants and microorganisms. Among them, nitrogen (N) can affect the fate of an interaction between a plant and a pathogen (Dordas, 2008). No general rules can be drawn about modification of resistance by N. Although we know that N lack or excess, along with the nature of available N in soil, can modulate plant resistance (Huber and Watson, 1974), the underlying mechanisms remain unclear. Recent works indicate that plants’ capacity to acquire and assimilate N could partly explain nutrition effects on plant defense. N is taken up by the roots mostly in the form of nitrate (NO_3_^-^) in aerobic soils and ammonium (NH_4_^+^) in flooded wetlands or acidic soils. Ammonium taken up directly from the soil or resulting from the reduction of NO_3_^-^ and nitrite (NO_2_^-^) by nitrate reductase (NR) and nitrite reductase (NiR), respectively, is assimilated *via* the glutamine synthetase (GS)/glutamate synthase cycle (Xu et al., 2012). The uptake of mineral N from the soil and the subsequent distribution to the whole plant is driven by nitrate transporters from the multigenic *NRT2* and *NPF* families and by ammonium transporters from the *AMT* family (Krapp, 2015). The contribution of several of these transporters to plant defense has recently been highlighted in *Arabidopsis thaliana*. For instance, induction of *AMT1.1* expression was evidenced upon infection by the bacterium *Pseudomonas syringae* or the fungus *Erysiphe cichoracearum* (Liu et al., 2010). The role of specific transporters was demonstrated using plant mutants: *nrt2* (deficient in the expression of the *NRT2.1* and *NRT2.2* genes) and *nrt2.6-1* mutants displayed altered sensitivity to the bacterial phytopathogens *P. syringae* and *E. amylovora* (Camanes et al., 2012; Dechorgnat et al., 2012). Besides N uptake into plants and its subsequent allocation, several results indicate that N assimilation and particularly amino acid homeostasis can impact plant-pathogen interactions (Zeier, 2013; Luna et al., 2014). Conversely, pathogen attacks are correlated with modulation of the expression of genes or of the activity of enzymes involved in N assimilation such as NR or GS2, in N remobilization such as GS1, and in amino acid metabolism [reviewed by Fagard et al. (2014)]. Whether these changes in N metabolism reflect the manipulation of host metabolism by the pathogen or result from the modulation of plant defenses is not always clear. Interestingly, some members of the GLR glutamate receptor family were recently proposed to play a role as amino acid sensors during plant defense, perhaps by sensing changes in extracellular amino acids caused by pathogen infection (Forde and Roberts, 2014). Crosstalk between N metabolism and phytohormones can also interfere with plant stress responses and could be considered as a mechanism involved in the partitioning of available resources between defense and growth. For instance, N limitation induced the accumulation of salicylic acid (SA) in *A. thaliana* leaves (Yaeno and Iba, 2008). Conversely, ethylene/jasmonic acid signaling coordinated the upregulation of the nitrate transporter *NRT1.8* (*AtNPF7.2*) and the downregulation of *NRT1.5* (*AtNPF7.3*) genes to tune NO_3_^-^ reallocation in plants from the shoot to the roots under stress conditions (Zhang et al., 2014). Finally, experiments on rice showed that N-induced susceptibility to *Magnaporthe oryzae* is genotype-dependent, and may be linked to N use efficiency (Ballini et al., 2013). These interesting data raise the question of the genetic control of N effects on plant immunity. The identification of the corresponding QTLs will permit to uncover new molecular actors of N-controlled resistance to pathogens.

## Nitric Oxide and N Metabolism

The role of NO in plant defense is widely accepted. NO is involved in transcriptional regulation of defense genes encoding PR proteins or proteins involved in phytoalexin synthesis, SA accumulation, and post-translational protein modifications (Wendehenne et al., 2014). NO is a nitrogen species produced *via* a variety of pathways in plants (reviewed by Gupta et al., 2011c). Briefly, these pathways can be classified into two groups according to nitrogen-containing precursors: the L-arginine-dependent pathway (oxidative pathway), and the NO_2_^-^-dependent pathway (reductive pathway). NO_2_^-^-dependent NO synthesis involves NR which reduces NO_2_^-^ to NO both *in vitro* and *in vivo* in specific physiological contexts (Yamasaki and Sakihama, 2000); alternatively, formation of NO through the reduction of NO_2_^-^ by the mitochondrial respiratory chain can also be observed, particularly in roots (Gupta et al., 2011a; Horchani et al., 2011). Finally, NO can be produced by an apoplastic non-enzymatic conversion of NO_2_^-^ to NO at acidic pH, in the presence of reductants such as ascorbic acid (Bethke et al., 2004).

Several pathways involved in NO transformation and turnover and balancing the bioavailability of this molecule have been identified (Leitner et al., 2009). Firstly, NO can react with reduced glutathione to produce *S*-nitrosoglutathione (GSNO), a low-molecular-weight *S*-nitrosothiol (SNO) that is more stable than NO and considered to be a mobile reservoir of NO. The cellular level of GSNO is enzymatically regulated primarily by GSNO reductase (GSNOR), which catalyzes the reduction of GSNO to oxidized glutathione and ammonium. Importantly, Yun et al. (2016) recently reported that NO and GSNO have additive functions in plant immunity but also in plant development. NO and GSNO might have distinct or overlapping molecular targets, thus allowing differential control of key cellular processes belonging to both defense and development. Secondly, besides their O_2_ binding properties, hemoglobins (Hbs) can metabolize NO into NO_3_^-^ and therefore are also considered as NO and NO_2_^-^ concentration modulators (Gupta et al., 2011b). Finally, NO rapidly reacts with superoxide (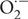) to form peroxynitrite (ONOO^-^), an oxidizing and nitrating RNS produced for instance in plant cells during immune responses (Vandelle and Delledonne, 2011). These molecules associated with NO turnover also play a role in the plant immune response. For instance, GSNO plays a key role in mediating the structural and functional changes of NPR1, a key transcription coactivator of plant immunity (Tada et al., 2008).

Nitric oxide is partly produced through NR, dependent on its substrate/product NO_3_^-^/ NO_2_^-^ as well as on L-arginine and polyamines. As a result, cross-regulation between NO signaling and N supply/metabolism is expected. Several lines of evidence show that NO production is likely to be affected by N supply. In a physio-pathological context, plant NO production is dependent on the form of N supply. Besson-Bard et al. (2008) and Gupta et al. (2013) showed that tobacco cell suspensions or leaves from plants grown on ammonium instead of nitrate as an N source emitted less NO when elicited by cryptogein or *P. syringae*. Thus these data highlight the determining role of the N source on the rate of NO synthesis. Modifications of the intracellular concentration of diverse intermediates of N metabolism such as amino acids or polyamines also result in the modulation of NO production. For instance, exogenously added polyamines induced rapid NO biosynthesis in *A. thaliana* (Tun et al., 2006). In the same manner, overexpression of the Asparagine synthetase 1 gene significantly enhanced the NO burst (Hwang et al., 2011). Finally, N nutrition could also impact important redox molecules associated with NO homeostasis. Nitrate deprivation led to altered levels of ROSs in *A. thaliana* and tobacco (Schachtman and Shin, 2007; Besson-Bard et al., 2008). Pathogen-induced expression of the nitrate transporter *NRT2.6* was also correlated with ROS accumulation (Dechorgnat et al., 2012). Concentrations of antioxidant molecules such as glutathione (GSH) were altered (decreased in shoots and increased in roots) in *A. thaliana* and barley plants exposed to N deficiency (Kandlbinder et al., 2004; Kovacik et al., 2014).

Reciprocally, NO and derived RNS could participate in the regulation of N metabolism. NO can control physiological processes by modifying gene transcription. By analyzing available literature and databases, we identified interesting candidates likely to contribute to the crosstalk between N metabolism and NO among the numerous NO-regulated genes. Transcriptomic studies highlighted the up- or down-regulation of transcripts encoding N transporters (Ahlfors et al., 2009; Corti Monzon et al., 2014; Trevisan et al., 2015) or N assimilation/remobilization genes (Ferrarini et al., 2008; Ahlfors et al., 2009; Xu et al., 2013; Begara-Morales et al., 2014; Corti Monzon et al., 2014; Zeng et al., 2014; Trevisan et al., 2015) and amino acid metabolism-related genes (Ferrarini et al., 2008; Xu et al., 2013) upon modulation of NO homeostasis by treatment with NO donors, NO scavengers, or using mutants affected in NO homeostasis. Physiological studies identified NO as a regulator of N uptake in *Chlamydomonas reinhardtii*, possibly through the control of the expression of the nitrate or ammonium (*AMT1.1 and AMT2.2*) transporters. In *A. thaliana*, the expression of the high affinity nitrate transporter *NRT2.1* was down-regulated by NO donors and in a *GSNOR* knock-out mutant, but the expression of the low-affinity nitrate transporter *NRT1.1* remained unaltered (Frungillo et al., 2014), suggesting a switch from high- to low-affinity nitrate transport. By contrast, the expression of *NRT2.1* was up-regulated through an NO-dependent process in *A. thaliana* roots exposed to cadmium (Besson-Bard et al., 2009). In addition to NO-mediated transcriptional regulation, many of NO biological functions arise as a direct consequence of chemical reactions between proteins and NO/RNS. Metal-nitrosylation, *S*-nitrosylation, and tyrosine nitration are notably emerging as main NO-dependent post-translational protein modifications (Astier and Lindermayr, 2012). Among the soluble proteins identified as *S*-nitrosylated or Tyr-nitrated, possible candidates contributing to the NO/N metabolism interplay are mainly involved in both N assimilation/remobilization and amino acid metabolism (**Table 1**). Post-translational inhibition of high-affinity ammonium and high-affinity NO_3_^-^/ NO_2_^-^ transporters by NO was highlighted in *C. reinhardtii* (Sanz-Luque et al., 2013). However, whether the reversible effect of NO was linked to *S*-nitrosylation of the transporters or to an indirect effect of NO leading to other post-translational modifications of the transporters remains to be determined (Sanz-Luque et al., 2013). In that same study, NO also inhibited NR activity reversibly, but not NiR or GS activity. This post-translational effect of NO on N transporters and NR might mediate the fast inhibition of N uptake and assimilation by ammonium in *C. reinhardtii*. More recently, inhibition of NR activity by NO was proposed to be partly mediated by a truncated hemoglobin THB1 whose gene expression is highly induced by NO (Sanz-Luque et al., 2015).

**Table 1:** Examples of *S*-nitrosylated or Tyr-nitrated proteins involved in N and amino acid metabolism.

Functions	Post-translational modifications	Identified Proteins	Conditions	Reference
Amino acid metabolism	Tyrosine nitration	Methionine synthase	–	Lozano-Juste et al., 2011
	*S*-nitrosylation	Asparagine synthase 3	Biotic stress	Maldonado-Alconada et al., 2011
		Glutamate decarboxylase	Biotic stress	Maldonado-Alconada et al., 2011
		EPSP synthase	Biotic stress	Astier et al., 2012
		Acetohydroxy acid isomeroreductase (Val and Ile synthesis)	Biotic stress	Astier et al., 2012
		Aspartate aminotransferase	Biotic stress	Astier et al., 2012
		Cysteine synthase	Abiotic stress	Puyaubert et al., 2014
		Alanine glyoxylate aminotransferase	Abiotic stress	Puyaubert et al., 2014
		Glutamate glyoxylate aminotransferase	Abiotic stress	Puyaubert et al., 2014
Nitrogen metabolism	Tyrosine nitration	Glutamine synthetase 2	Biotic stress	Cecconi et al., 2009; Lozano-Juste et al., 2011
		Glutamine synthetase 1	Rhizobium-legume symbiosis	Melo et al., 2011
	*S*-nitrosylation	Argininosuccinate synthase	Biotic stress	Maldonado-Alconada et al., 2011
		Nitrite reductase	*atgsnor1–3*	Hu et al., 2015
		Glutamate synthase	Abiotic stress	Puyaubert et al., 2014
		Glutamate dehydrogenase 1	Biotic stress	Maldonado-Alconada et al., 2011
		Glutamate dehydrogenase 2	Biotic stress	Maldonado-Alconada et al., 2011


In higher plants, NO produced by denitrification in the rhizosphere of forest soils impacts N uptake without affecting gene expression patterns of putative N transporters, suggesting post-translational modification of these transporters (Dong et al., 2015). NR is also highly regulated by complex transcriptional and post-translational mechanisms. Studies on different models using NO donors, NO synthase inhibitors, or the scavenger cPTIO indicate that NO modulates NR activity. Results are sometimes contradictory. NR activity in leaves was inhibited under high NO concentrations (Rosales et al., 2011, 2012; Frungillo et al., 2014), but was enhanced in cabbage (Du et al., 2008). Moreover, the inhibition or activation of NR by NO in tomato roots could depend on the NO_3_^-^ concentration (Jin et al., 2009). The mechanisms explaining these effects of NO on NR are poorly understood. Regulation of NR by NO could occur through transcriptional downregulation of the NR *NIA* genes in *Chlamydomonas* and *A. thaliana* (de Montaigu et al., 2010). A direct interaction of NO with NR is possible, as *S*-nitrosylation of NR was evidenced in poplar exposed to cold stress (Cheng et al., 2015). Glutamine synthetase 2 is a second key enzyme of plant N metabolism involved in the synthesis of essentially nitrogenous compounds *via* Gln production. Interestingly, GS1 and GS2 were identified as molecular targets of NO (**Table 1**). GS activity was inhibited by Tyr nitration in root nodules of *Medicago truncatula*. This post-translational modification may mediate channeling of glutamate to boost plant antioxidant defenses (Melo et al., 2011) in response to NO. This interesting feature does not seem to be shared across the plant kingdom since GS activity was not affected by the NO donor DEA-NONOate in the alga *Chlamydomonas* (Sanz-Luque et al., 2013).

## Role of NO/RNS in the Modulation of the Immune Response by N Nutrition: First Experimental Evidence

Altogether, these data indicate that N supply has an impact on plant immunity and NO/RNS signaling and lead us to wonder about the role of NO/RNS in the modulation of the immune response by N nutrition. In the present work, we used an *in vitro* pathosystem composed of the legume *M. truncatula* challenged with the soil-borne root pathogen *Aphanomyces euteiches*. This oomycete is considered as the most limiting factor for legume production. Resistance of *M. truncatula* roots includes protection of the central cylinder against pathogen invasion, associated with frequent pericycle cell divisions, lignin deposition, and accumulation of soluble phenolic compounds (Djébali et al., 2009). First investigations of the biochemical processes underlying the expression of this resistance showed modulation of H_2_O_2_ levels and of the activity of antioxidant enzymes (Djébali et al., 2009, 2011). Interestingly, in the *M. truncatula* A17 genotype, resistance against *A. euteiches* was significantly enhanced in response to NO_3_^-^ starvation as compared to sufficient N conditions (Thalineau et al., unpublished). Based on the current literature, we hypothesized that NO could play a role in this N-induced modulation of *M. truncatula* defense responses against *A. euteiches*. We therefore first assessed whether changes in NO homeostasis could indeed affect *M. truncatula* resistance to *A. euteiches*. Secondly, we determined whether NO homeostasis could be modulated by N nutrition during the *M. truncatula-A. euteiches* interaction. We considered NO homeostasis as the maintenance of a functional NO concentration in a specific condition, through a balance between its biosynthesis (e.g., NR activity) and turnover pathways (e.g., interactions with GSH or O2^⋅-^ to form GSNO or ONOO^-^, respectively).

## Materials and Methods

### Plant Growth and Inoculation by *A. euteiches*

We used the *M. truncatula* Jemalong-A17 genotype. *M. truncatula* seeds were scarified according to Djébali et al. (2009). After stratification overnight at 4°C, they were germinated in phytochambers with 16 h light under 350 μmol m^-2^ s^-1^ photons at 23°C/8 h night at 21°C. One day after germination, the seedlings were transferred to 12 cm × 12 cm square Petri dishes containing modified M medium (Bécard and Fortin, 1988). This modified medium was sugar-free, enriched in phosphate (1.3 mM final concentration), and contained either 3.2 mM nitrate (complete medium) or no nitrate (NØ medium). The Petri dishes were sealed with parafilm and the roots were protected from light with aluminum foil, and then placed vertically in the culture chamber (16 h light under 350 μmol m^-2^ s^-1^ photons at 23°C/8 h night at 21°C) for 7 days. The strain *Aphanomyces euteiches* Drechs ATCC 201684 was used to inoculate the seeds one day after germination. Zoospores were produced as described in Rey et al. (2013), and each root was inoculated with 500 zoospores.

### *Agrobacterium rhizogenes* Root Transformation

The pENTR4 vector carrying the MtNR1 or the MtNR2 fragment (Horchani et al., 2011) was recombined with the pK7GWIWG2d vector using LR clonase II enzyme mix (Invitrogen, France) to create RNA interference expression vectors. The MtGSNOR gene (*M. truncatula* Gene code Medtr7g099040) (1,143 bp) was amplified using *M. truncatula* cDNA as a template and the specific primers GSNOR-F 5′-AAAAAGCAGGCTTCACCATGGCATCGTCGACTGAAGGT-3′ and GSNOR-R 5′- AGAAAGCTGGGTGTCAATGCAATGCAAGCACAC containing the corresponding attB recombination sites. The PCR product was recombined into the pDONR entry vector (Invitrogen) and checked by sequencing. The pDONR vector carrying the MtGSNOR gene was recombined with pK7WG2d plasmids^1^ to create the overexpression vector. The constructs pK7GWIGW2d-MtNR1-2/GFP (RNAi::*MtNIA1/2*) and pK7WG2d-MtGSNOR/GFP (35S::GSNOR) were introduced into *A. rhizogenes* strain Arqua1 (Quandt et al., 1993). *M. truncatula* plants were transformed with *A. rhizogenes* according to Boisson-Dernier et al. (2001). Control plants were transformed with *A. rhizogenes* containing the pK7GWIGW5D or the pK7WG2d empty vectors. Hairy roots were selected based on the fluorescent marker GFP 21 days after transformation.

### RNA Extraction, Reverse Transcription, and Quantitative PCR on Transformed Roots

Total RNA was extracted from transformed roots using TRIzol^®^ Reagent (Life Technologies) according to the manufacturer’s recommendations. To carry out the qPCR reaction, RNAs (0.5–1 μg) were reverse-transcribed in a final volume of 20 μL in the presence of RNasin (Promega, Charbonnières, France), and oligo(dT)_15_, with M-MLV reverse transcriptase (Promega, Charbonnières, France), as recommended by the manufacturer.

Quantitative PCR was performed on reverse-transcribed RNAs from four independent biological replicates per condition and from two independent plant cultures. Quantitative PCR reactions were performed in an ABI PRISM 7900 sequence detection system (Applied Biosystems^®^, Saint-Aubin, France), in a final volume of 15 μL containing Absolute SYBR green ROX Mix (Thermo Scientific, Surrey, UK), 0,3 μM of gene-specific primers, and 5 μL of cDNA template diluted 60-fold. The reference gene used for normalization was *MtEF1α.* Relative expression was expressed as 2^-ΔCt test genes-reference gene^. The primers used for the qPCR all displayed a high amplification efficiency (90–100%). They were the following:

MtGSNORforward 5′-GTGACTGGGCGTGTATGGAA-3′
MtGSNORreverse 5′-TGCAAGCACACAACGAAGAC-3′
MtNIA1forward 5′-TGTTCCACAGGCTTCTCCAGATACA-3′
MtNIA1reverse 5′-CATACAGCGTCGTACTCAGCGACA-3′
MtNIA2forward 5′GCAAACCGGACGGAGGATGA-3′
MtNIA2reverse 5′CCGTGATGAATCCCACACTATATTCC-3′
MtEF1αforward 5′-AAGCTAGGAGGTATTGACAAG-3′
MtEF1αreverse 5′-ACTGTGCAGTAGTACTTGGTG-3′

### Inoculation of Transformed Root Cultures with *A. euteiches*

Roots were cultured on Shb10 medium (Boisson-Dernier et al., 2001) and transferred on modified Fahraeus medium enriched in ammonitrate (1 mM NH_4_NO_3_ final) one day before inoculation. Inoculation of the root cultures with *A. euteiches* strain ATCC 201684 was carried out by adding 10 mL of an *A. euteiches* zoospore suspension containing 80,000 zoospores.mL^-1^ in sterilized Volvic (Colditz et al., 2007) water. Zoospore production was initiated as described in Rey et al. (2013). Control root cultures were inoculated with 10 mL of sterile Volvic water. After 4 h of incubation in the dark, the zoospore solution was drained off the roots, and the Petri dishes were placed back into the growth room and left there for 7 days in the dark.

### Assessing Infection Levels by Enzyme-Linked Immunosorbent Assay (ELISA)

Assessment of *A. euteiches* development in roots was performed by ELISA, using rabbit polyclonal serum raised against *A. euteiches*, and a mouse anti-rabbit IgG alkaline phosphatase conjugate as described by Slezack et al. (1999), on protein extracts from roots from pooled plants. Alkaline phosphatase activity was monitored by recording the increase in absorbance at 405 nm for 2–3 h, and was expressed as the slope of the resulting curve per mg of root fresh weight.

### Hydrogen Peroxide Quantification

H_2_O_2_ concentration was measured using an Amplex Red^®^/peroxidase-coupled fluorescence assay adapted from Ashtamker et al. (2007). Roots were ground on ice and in the dark, in 1 mL of KRPG buffer (145 mM NaCl; 5.7 mM K_2_HPO_4_; 4.86 mM KCl; 0.54 mM CaCl_2_; 1.22 mM MgSO_4_; 5.5 mM glucose; pH 7.35) with 10 μM Amplex Red^®^ and 0.2 U/mL of Horse Radish Peroxidase (HRP) per 100 mg of fresh weight. Catalase, an H_2_O_2_ scavenger, was used as a control. After 10 min of incubation at 4°C with catalase (1 unit/μL), 10 μM Amplex Red^®^ and 0.2 U/mL of HRP were added to the samples. After centrifugation (10,000×*g*, 15 min, 4°C), 100 μL of supernatant were used to quantify resorufin (λ_ex_ = 560 nm; λ_em_ = 584 nm) by spectrofluorimetry (Mithras, Berthold Technology). The relative fluorescence units were converted into μmol of H_2_O_2_ mg^-1^ root fresh weight on the basis of a standard curve established from known concentrations of H_2_O_2_.

### Nitric Oxide and Peroxynitrite Quantification

ONOO^-^ and NO concentrations were determined using A17 or transformed roots ground on ice and in the dark, with 1 mL of Tris-HCl (10 mM, pH 7.5), KCl (10 mM) buffer with 5 μM aminophenyl fluorescein (APF) or 10 μM 4,5-diaminofluorescein (DAF), respectively, per 100 mg of fresh weight. Epicatechin, an ONOO^-^ scavenger, was used as a control. After 10 min of incubation at 4°C with epicatechin (1 mM), APF was added to the samples at a final concentration of 5 μM. cPTIO, an NO scavenger, was used as a control. After 10 min of incubation at 4°C with cPTIO (500 μM), DAF was added to the samples at a final concentration of 10 μM.

After centrifugation (10,000×*g*, 15 min, 4°C), 100 μL of supernatant were used to quantify ONOO^-^ or NO (λ_ex_ = 485 nm; λ_em_ = 535 nm) by spectrofluorimetry (Mithras, Berthold Technology).

### S-nitrosothiol Quantification

*S*-nitrosothiol quantification was performed using the Saville–Griess assay (Gow et al., 2007). A17 roots or transformed roots were ground, on ice and in the dark, in extraction buffer (1 mL/100 mg of fresh weight, 0.1 M Tris-HCl, pH 7.5; 1 mM PMSF). After centrifugation (10,000×g, 15 min, 4°C), 100 μL of supernatant were incubated with 100 μL of buffer *A* (0.5 M HCl; 1% sulfanilamide) or 100 μL of buffer *B* (0.5 M HCl; 1% sulfanilamide; 0.2% HgCl_2_). After incubation (15 min at room temperature), 100 μL of Griess reagent[(0.5 M HCl; 0.02% N-(1-naphtyl)-ethylenediamine dihydrochloride] were added. After 15 min, SNOs were quantified by measuring absorbance at 540 nm. A standard curve was obtained using different concentrations of GSNO.

### Nitrate Determination

Nitrate determination was performed according to Miranda et al. (2001), based on the reduction of nitrate to nitrite by vanadium and colorimetric detection at 540 nm of nitrite in the presence of sulfanilamide and N-1-naphthylethylenediamine. Approximately 100 mg of 7-day-old plant roots were collected, flash-frozen in liquid N_2_, and ground into powder. Three hundred micro liter of ultra-pure water were added to 20 mg of frozen sample, thoroughly vortexed, and incubated with occasional mixing for 15 min on ice. After centrifugation 15 min at 13,000×*g* and 4°C, the supernatant was recovered and used for nitrate determination.

### Nitrate Reductase Activity Measurements

Transformed root samples were frozen in liquid nitrogen and ground using pestle and mortar. Extraction was performed in MOPS buffer (1 mL per 100 mg of fresh weight, 50 mM MOPS-KOH buffer, pH 7.6; 5 mM NaF; 1 μM Na_2_MoO_4_; 10 μM FAD; 1 μM leupeptin, 0.2 g/g FW polyvinylpolypyrrolidone; 2 mM β-mercaptoethanol; 5 mM EDTA). After centrifugation (20,000×*g*, 5 min, 4°C), the supernatant was used to measure NR activity. The reaction mixture consisted of 50 mM MOPS-KOH buffer, pH 7.6, containing 1 mM NaF, 10 mM KNO_3_, 0.17 mM NADH, and 5 mM EDTA. After incubation 15 min at 30°C, the reaction mixture was stopped by adding an equal volume of sulfanilamide (1% w/v in 3 N HCl) followed by N-naphtylethylenediamine dihydrochloride (0.02%, w/v), and the A_540_ was measured. A standard curve was obtained based on different concentrations of nitrite.

### GSNOR Activity Measurements

To measure GSNOR activity, roots were ground in liquid nitrogen and proteins were extracted in 50 mM Tris-HCl buffer, pH 8, 0.5 mM EDTA, and 1 mg/mL of a protease inhibitor cocktail (1 mL of buffer per 100 mg of fresh weight). GSNOR activity was assayed from the rate of NADH oxidation by measuring the decrease in absorbance at 340 nm at 25°C using 25 μg of proteins in a total volume of 200 μL of extraction buffer containing 350 μM NADH with or without 350 μM GSNO. GSNO reductase activity was determined by subtracting NADH oxidation values in the absence of GSNO from values in the presence of GSNO. All samples were protected from light during the assay and tested for linearity. A standard curve was obtained using different concentrations of NADH.

### Statistical Analyses

Statistical analyses were performed using one- or two-way analysis of variance (ANOVA) followed by Fisher’s test. Data were considered as significantly different when *p* < 0.05.

## Results and Discussion

### NO Homeostasis Participates in the *M. truncatula* Immune Response

**FIGURE 1:**
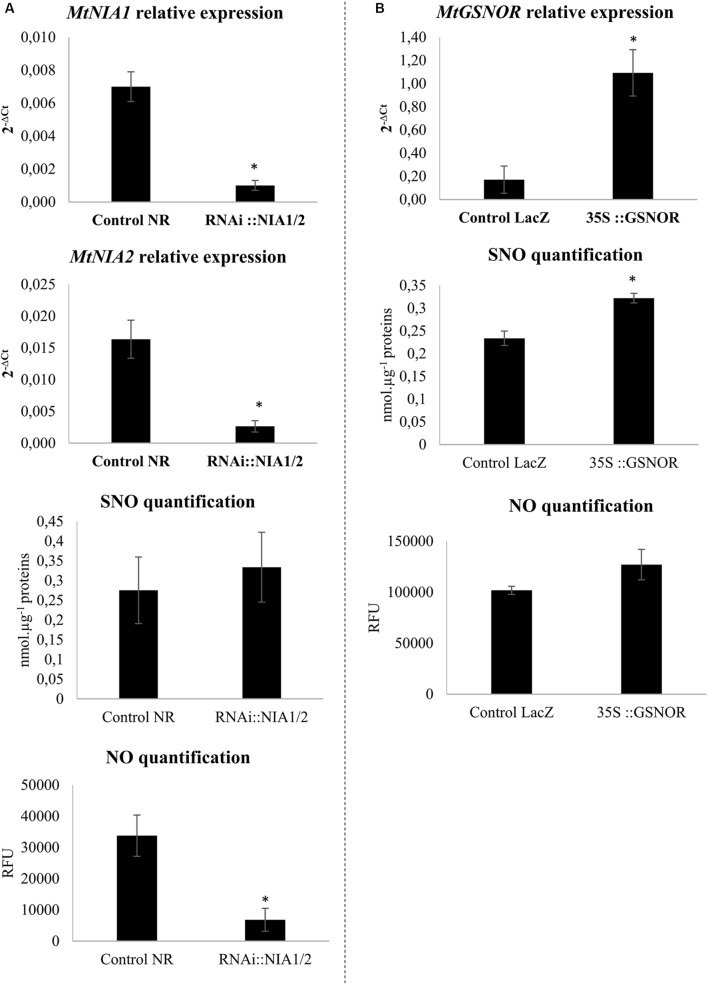
**Transformed root validation**. **(A)** Transcript levels of *MtNIA1* and *MtNIA2* in *RNAi::NIA1/2-*transformed roots were compared to control transformed roots (control NR). SNO quantification using the Saville–Griess assay and NO quantification using the fluorophore DAF (10 μM). Control NR and *RNAi::NIA1/2*-transformed roots extracts were pre-incubated or not with 500 μM cPTIO as an NO scavenger. **(B)** Transcript levels of *MtGSNOR* in *35S::GSNOR-*transformed roots were compared to control transformed roots (control LacZ). SNO quantification using the Saville–Griess assay and NO quantification using the fluorophore DAF (10 μM). Control LacZ and *35S::GSNOR-*transformed roots extracts were pre-incubated or not with 500 μM cPTIO as an NO scavenger. Error bars indicate standard errors (*n* = 4 for transcripts and NO levels; *n* = 8 for SNO levels), and ^∗^ indicates significant differences (*p* < 0.05).

To investigate the putative role of NO homeostasis in the *M. truncatula/A. euteiches* interaction, roots were transformed to inactivate the NR-encoding *MtNIA1/2* genes or to overexpress GSNOR-encoding genes. Quantification of gene transcripts in transformed roots using RT-qPCR confirmed that the two *NIA* genes were repressed (**Figure 1A**) while *GSNOR* was overexpressed (**Figure 1B**). To perform functional validation of the different constructs, we quantified NO and SNO levels in transformed roots. The two genetic manipulations modulated NO or SNO levels (**Figure 1**). SNO levels remained unchanged in *RNAi::MtNIA1/2* roots as compared to the controls, whereas NO levels clearly decreased (**Figure 1A**). This was in accordance with the downregulation of NR, a major enzymatic source of NO. Conversely, NO levels in the *35S::GSNOR* roots did not significantly change, but SNO significantly increased as compared to control roots (**Figure 1B**). This was surprising because in most previous experiments a negative correlation was described between SNO levels and GSNOR activity (Feechan et al., 2005; Rusterucci et al., 2007; Yun et al., 2011). However, it is interesting to note that in pea (a legume closely related to *M. truncatula*), higher SNO levels induced by wounding were correlated with higher GSNOR activity (Corpas et al., 2008).

**FIGURE 2:**
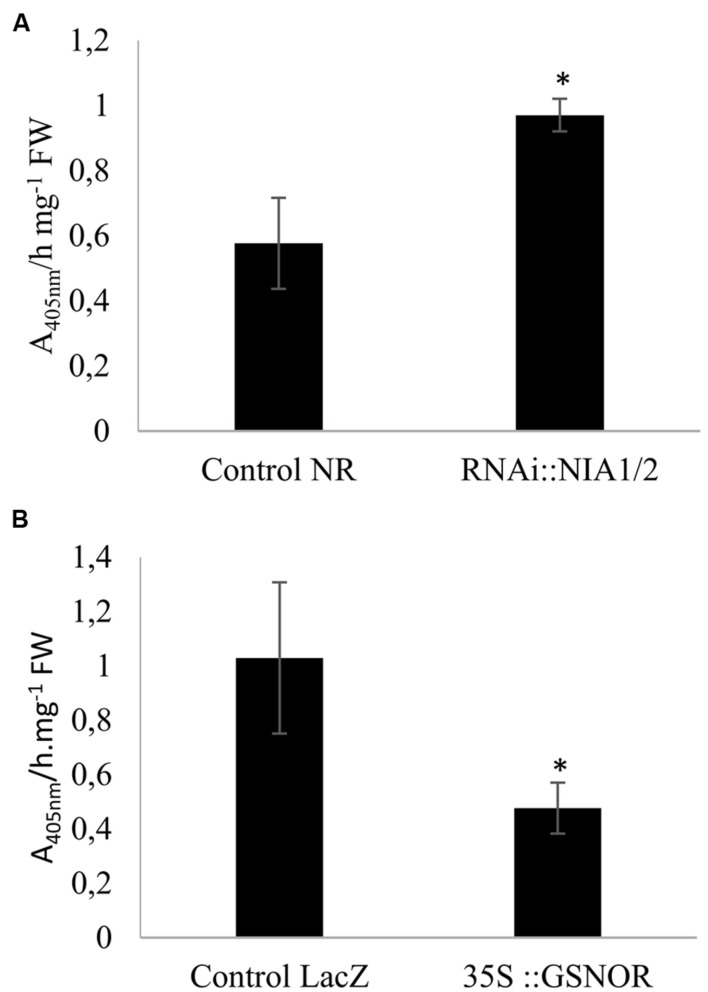
**Quantification of *Aphanomyces euteiches* in extracts from inoculated transformed roots.** *RNAi::NIA1/2*-transformed roots **(A)** and *35S::GSNOR*-transformed roots **(B)** were extracted for ELISA tests. Roots were cultivated *in vitro* for 7 days on Fahraeus medium and then inoculated with *A. euteiches*. The background signal in non-inoculated roots was subtracted from the signal detected in inoculated roots. Error bars indicate standard errors (*n* = 4), and ^∗^ indicates significant differences (*p* < 0.05). Data from one representative experiment out of four independent experiments.

We studied the impact of these genetic transformations on the *M. truncatula/A. euteiches* interaction. ELISA tests using antibodies raised against *A. euteiches* (Slezack et al., 1999) were performed to quantify the presence of the pathogen in roots. In *RNAi::MtNIA1/2* roots (**Figure 2A**), *A. euteiches* colonization was significantly greater than in control transformed roots (Control NR roots). These data reaffirm the role of the NR enzyme in the plant immune response. In *A. thaliana*, the NR-deficient double mutant (*nia1 nia2*) failed to exhibit a hypersensitive response and was hyper-susceptible to *P. syringae* (Modolo et al., 2006; Oliveira et al., 2009) and to the necrotrophic fungal pathogens *Sclerotinia sclerotiorum* or *Botrytis cinerea* (Perchepied et al., 2010; Rasul et al., 2012). Although these effects were attributed to the substantially reduced NO levels in this mutant, a side effect of N metabolism on plant defense cannot be excluded as NR stands at the crossroads between N metabolism and NO production.

Our results using *GSNOR*-transformed roots showed that pathogen levels were lower in *GSNOR*-overexpressing roots (**Figure 2B**) than in control transformed roots (Control *LacZ* roots). GSNOR could therefore be considered as a positive regulator of *M. truncatula* resistance to *A. euteiches*. Previous works already investigated the physiological roles of GSNOR in plant-pathogen interactions, using transgenic *A. thaliana* plants (Feechan et al., 2005; Rusterucci et al., 2007; Yun et al., 2011). Results are sometimes contradictory, as modulation of *AtGSNOR* expression enhanced or decreased plant disease resistance depending on the pathosystem. GSNOR could play a significant role in plant immunity because GSNO is considered as a mobile reservoir of NO, is more stable than NO, and is a transnitrosylation agent of proteins. The contrasted results obtained in our study with *NR* and *GSNOR* constructs could be attributed to the specific roles of the corresponding proteins in NO homeostasis. NR is involved in NO synthesis, whereas the primary role of GSNOR is to regulate GSNO contents. The recent results from Yun et al. (2016) confirm that GSNO and NO may play distinct roles in plant immunity by acting on different molecular targets. In addition, GSNOR indirectly affects NO, GSH, ROS, and total intracellular nitrosothiol (SNO) levels, indicating that GSNOR might be more globally involved in the regulation of the cell redox state (Espunya et al., 2006; Yun et al., 2011).

**FIGURE 3:**
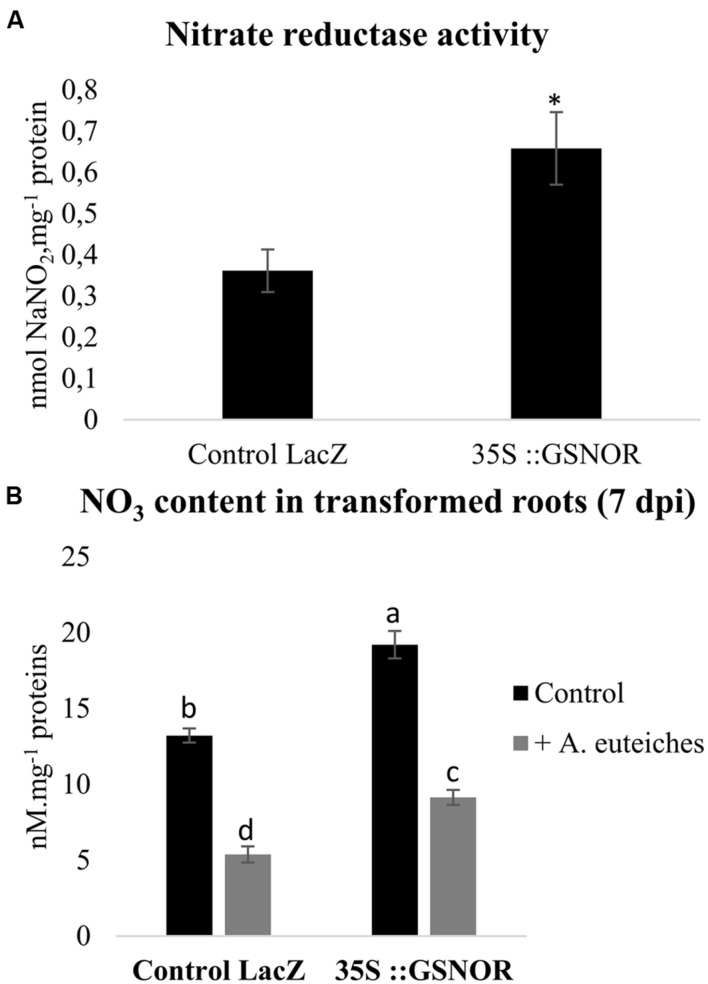
**Nitrate reductase (NR) activity and NO_3_^-^ contents in transformed roots.** **(A)** NR activity in control transformed roots (Control LacZ roots transformed with pK7GWG2D-GFP) and in transformed roots overexpressing GSNOR (*35S::GSNOR*). Transformed roots were cultivated *in vitro* on Shb10 medium. **(B)** NO_3_^-^ concentrations in control transformed roots (Control LacZ) and *GSNOR*-overexpressing roots. Transformed roots were cultivated *in vitro* for 7 days on Fahraeus medium, and inoculated with *A. euteiches.* Error bars indicate standard errors (*n* = 4), and letters or ^∗^ indicate significant differences (*p* < 0.05). Data from one representative experiment out of three independent experiments for both NR activity and NO_3_^-^ contents (*n* = 12).

Nitric Oxide partly regulates N metabolism. Therefore we also investigated the effects of *GSNOR* overexpression on root NO_3_^-^ contents and NR activity in transformed roots. *GSNOR* overexpression increased basal NO_3_^-^ content and NR activity (**Figures 3A,B**). Modulation of N metabolism by GSNO and NO in *A. thaliana* has been described (Frungillo et al., 2014), and was explained by the effect of NO and GSNO on NR activity and on the expression of the *AtNRT2.1* high-affinity NO_3_^-^ transporter gene. Similarly to our data, that study shows that *GSNOR* overexpression is correlated with higher NR activity and NO_3_^-^ content. Interestingly, we noted that pathogen colonization reduced NO_3_^-^ concentrations in roots by approximately 65%, suggesting an effect of *A. euteiches* on nitrate transport and/or NO_3_^-^ assimilation. Although we found a higher NO_3_^-^ content in 35S::GSNOR-infected roots than in control infected roots, the amplitude of the pathogen-induced decrease in NO_3_^-^ level was not impacted in *35S::GSNOR* roots, suggesting that this process is independent of GSNO homeostasis. This reduced level of NO_3_^-^ is unlikely to result from consumption of NO_3_^-^ by the pathogen: data mining of the *A. euteiches* database revealed that no homologs of the NR, NIR, and NO_3_^-^ transporter (NRT2) genes were detected in the genome of this pathogen^2^, confirming earlier observations that NO_3_^-^ is unfavorable for *A. euteiches* development (Huber and Watson, 1974). Alternatively, we cannot exclude that the decreased NO_3_^-^ content in infected roots could be due to nitrate leakage from the roots related to developing necrosis.

### Effect of N Nutrition on NO/ H_2_O_2_/ONOO^-^ Accumulation and SNO Contents

**FIGURE 4:**
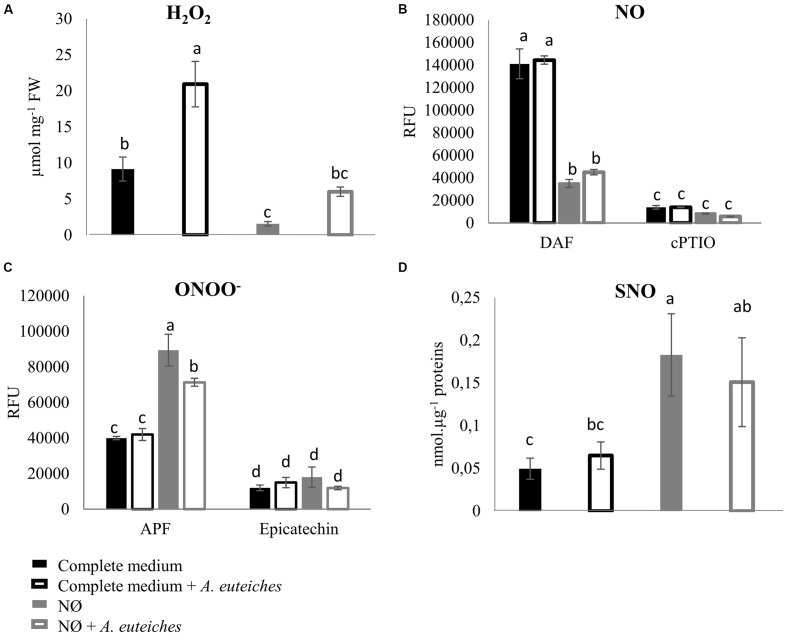
**H_2_O_2_, NO, ONOO^-^, and SNO quantification in *Medicago truncatula* roots 7 dpi.** *M. truncatula* was cultivated on complete medium or NØ medium, and roots were harvested 7 days after inoculation with *A. euteiches* and used to detect H_2_O_2_, NO, and ONOO^-^ concentrations using fluorescent probes, and SNO concentrations using the Saville–Griess assay. **(A)** H_2_O_2_ quantification using 10 μM Amplex Red^®^ fluorophore and 0.2 U/mL of peroxidase. Catalase (1 U/μL), used as an H_2_O_2_ scavenger, abolished Amplex Red^®^ fluorescence. **(B)** NO quantification using the fluorophore DAF (10 μM). Root extracts were pre-incubated or not with 500 μM cPTIO as an NO scavenger. **(C)** ONOO^-^ quantification using the fluorophore APF (5 μM). Root extracts were pre-incubated or not with 1 mM of the ONOO^-^ scavenger epicatechin. **(D)** SNO quantification by the Saville–Griess assay. Error bars indicate standard errors (*n* = 4 for **A–C**; *n* = 14 for **D**), and letters indicate significant differences (*p* < 0.05). Data from one representative experiment out of three independent experiments for H_2_O_2_, NO, and ONOO^-^concentrations, and data corresponding to two independent experiments pooled together for SNO concentrations. RFU, relative fluorescence units.

To analyze the role of N availability on NO, H_2_O_2_, and ONOO^-^ accumulation, *M. truncatula* plants were cultivated in complete medium or NO_3_^-^-deficient medium (NØ), and inoculated or not with *A. euteiches*. The NO scavenger cPTIO and the ONOO^-^ scavenger epicatechin were used as controls to check the specificity of the fluorescence probes. We observed that NO_3_^-^ deficiency caused a significant increase in ONOO^-^ content on NØ medium (**Figure 4C**), whereas NO and H_2_O_2_ levels decreased (**Figures 4A,B**), highlighting a link between NO_3_^-^ content and production of these reactive species. A clear effect of pathogen colonization was only evidenced for H_2_O_2_ contents (**Figure 4A**), and this increase was abolished on NØ. Surprisingly, although NO production is considered as a common response to pathogens, no increase in NO levels was detected in response to *A. euteiches* (**Figure 4B**). More generally, whereas NO, ROS, or ONOO^-^ production has been widely described in response to pathogens, the literature does not give a clear picture of the cross-talks between these molecules. For instance, we observed a negative correlation between NO and ONOO^-^ contents in response to NO_3_^-^ deficiency, but in other models high NO levels are often correlated with high ONOO^-^ levels (Abramowski et al., 2015; Kulik et al., 2015). These conflicting observations raise some questions. Are these discrepancies due to plant models or due to the difficulty in measuring and precisely localizing these molecules? Differences in the stability of these molecules or their specific scavenging by plants during pathogen attack could explain why we did not detect changes in ONOO^-^ or NO levels in response to *A. euteiches*. Moreover, NO could also be used by the pathogen to activate its own metabolism, an important step in plant infection by fungi (Sedlářová et al., 2016).

**FIGURE 5:**
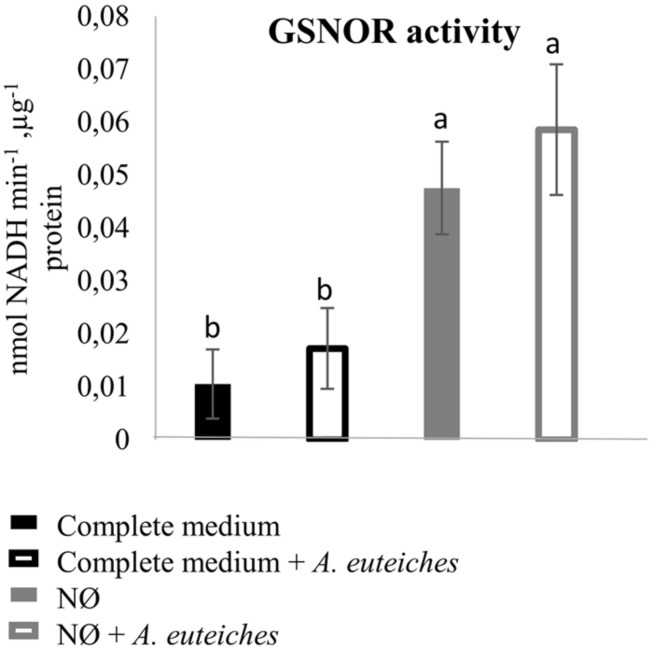
**GSNO reductase activity in *M. truncatula* roots 7 dpi.** *M. truncatula* seedlings were inoculated or not with *A. euteiches*, and cultivated on complete medium or NØ medium for 7 days. Root extracts were used to measure GSNOR activity. Error bars indicate standard errors (*n* = 4), and letters indicate significant differences (*p* < 0.05). Data from one representative experiment out of four independent experiments.

We also measured root SNO levels and GSNOR activity in the biological conditions of interest. Root SNO contents, determined using the Saville–Griess method, significantly increased on NØ medium as compared to the complete medium (**Figure 4D**). In response to *A. euteiches*, no significant change in SNO levels was highlighted (**Figure 4D**). Therefore, on NØ medium, the SNO content evolved in an opposite way to the NO content, similarly to the ONOO^-^ content. This result is in accordance with results reported in *Helianthus annuus* (Chaki et al., 2011), and can be attributed to the fact that NO is the source for ONOO^-^ and SNO. By contrast, a high NO content can be correlated with a high SNO content when plants are grown on culture medium containing NO_3_^-^ (Abramowski et al., 2015; Pietrowska et al., 2015). Our data also suggest that NO_3_^-^ nutrition impacts the overall balance between NO, ONOO^-^, and SNO. Regarding GSNOR, no changes in its activity was detected upon inoculation, in line with the absence of change in SNO levels in infected roots. In the roots of plants cultivated on NØ (**Figure 5**), higher GSNOR activity was correlated with higher SNO levels, confirming the positive correlation between GSNOR activity and SNO levels observed in 35S::GSNOR-transformed *Medicago* roots (**Figure 1**) and in pea, a closely related legume (Corpas et al., 2008). The positive or negative correlation between GSNOR activity and SNO levels or between NO and SNO levels depending on plant species and experimental conditions can be explained by several hypotheses. The SNO level is regulated through nitrosylation and denitrosylation; GSNOR, by controlling the level of GSNO, indirectly affects the level of *S*-nitrosylation. However, the TRX (thioredoxin)/NTR (NADPH-dependent TRX reductase) enzymatic system also controls *S*-nitrosylation (Kneeshaw et al., 2014). Interestingly, these activities were also identified in roots and activated by NO, leading to denitrosylation of specific proteins (Correa-Aragunde et al., 2015). Thus, these results, together with our study, illustrate the complex relationships between NO production/GSNOR activity and total SNO levels. Abiotic stresses also increase GSNOR activity (Kubienova et al., 2014), and this appears to be the case for *M. truncatula* plants under NO_3_^-^ deficiency. Higher GSNOR activity in N-deprived roots (**Figure 5**) could lead to a physiological state inducing higher resistance to *A. euteiches*, as observed in the 35S::GSNOR-transformed roots (**Figure 2B**). This could partly explain the enhanced resistance to this oomycete on NØ medium despite the low levels of NO in the roots. Thus, altogether our data highlight the possible positive and non-redundant roles of NO (**Figures 1A** and **2A**) and SNO (**Figures 1B**, **2B**, and **4D**) in mediating *M. truncatula* resistance to *A. euteiches*.

## Conclusion and New Hypotheses

**FIGURE 6:**
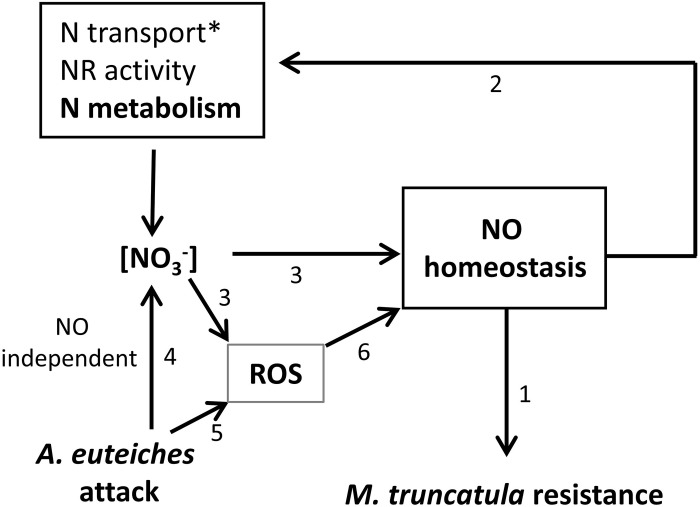
**Working model.** Results from the present work indicate that *RNAi::MtNIA1/2* and *35S::GSNOR* transformed roots are, respectively, more susceptible and more resistant to *A. euteiches* (1). NR activity and NO_3_^-^ content were impacted by GSNOR overexpression, indicating a possible effect of GSNOR on basal NO_3_^-^ transport/assimilation (2). NO_3_^-^ availability in the medium causes quantitative modulation of ROS/RNS/NO content and affects their balance (3). Infection by *A. euteiches* decreases root NO_3_^-^ content (4) and induces higher ROS levels (5). According to the literature superoxyde (O_2_^⋅-^), by reacting with NO to form peroxynitrite, can influence the concentration of NO available for signaling (6). ^∗^: GSNO was shown to regulate NO_3_^-^ uptake through transcriptional regulation of NRT2.1 (Frungillo et al., 2014).

The results obtained in the present study are summarized in **Figure 6**. We have demonstrated, using transformed roots affected in genes involved in NO synthesis (*NIA* genes) and turnover (GSNOR gene), that deregulation of NO homeostasis has an effect on *M. truncatula* resistance against *A. euteiches*, as observed in other pathosystems (1). In addition, it appears that the modulation of NO homeostasis (through GSNOR overexpression) impacts NR activity and NO_3_^-^content, indicating possibly an effect of GSNOR (or GSNO) on basal NO_3_^-^ transport/assimilation and confirming the results of Frungillo et al. (2014) (2). In return, NO_3_^-^ availability in the medium can affect NO homeostasis by modulating ROS/RNS/NO contents and their balance (3). Finally, infection by *A. euteiches* decreases root NO_3_^-^ content (4) and induces higher ROS levels (5). Altogether these results highlight the close interplay occurring between N nutrition and NO homeostasis as well as the involvement of NO in the modulation of plant resistance by N nutrition.

Future work should take into account the role of N availability on NO-mediated plant molecular responses. Thus, the study of the specific role of GSNO in this process through the identification *S*-nitrosylated/denitrosylated proteins under different N availability conditions and N sources seems promising. A focus will be made on proteins involved in the plant immune response (1), but also on the feedback regulation of N metabolism by NO because NO could control NO_3_^-^ availability and therefore plant resistance (2) (**Figure 6**). Investigations using foliar pathogens and other plant models will lead to a possible generalization of this phenomenon. More generally, plant N use efficiency can be affected by NO since NO controls not only N metabolism but also plant root growth and architecture changes in response to NO_3_^-^ (Manoli et al., 2014; Sun et al., 2015). Recent data show that plant N use efficiency and N-induced susceptibility to pathogens may be linked (Ballini et al., 2013). Consequently future studies should also focus on candidate proteins involved in root development. Finally, experiments conducted with plant genotypes differing in their resistance levels will permit to study the quantitative effect of NO/ROS production on plant defense.

## Author Contributions

ET, H-NT, ABo, and SJ conceived and designed the research; ET, H-NT, CF, ABo, and ABe carried out the experiments and analysis/interpretation of data; ET, H-NT, DW, and SJ wrote the manuscript. All authors contributed to the discussion and approved the final manuscript.

## Conflict of Interest Statement

The authors declare that the research was conducted in the absence of any commercial or financial relationships that could be construed as a potential conflict of interest.

## Footnotes

1 https://gateway.psb.ugent.be/

2 http://www.polebio.lrsv.ups-tlse.fr/aphano/
